# Supplementary material for: Are carbon emissions trading and green financial instruments synergistic? -Comprehensive quantitative research based on content analysis
Source: PLoS One. 2024 Mar 7;19(3):e0298601. doi: 10.1371/journal.pone.0298601 (PMC10919601; doi:10.1371/journal.pone.0298601)
Supplement: S1 Data — (ZIP) [file pone.0298601.s001.zip › Data/地级市Z_F工作报告.zip!/░▓╗╒/╗╞╔╜╩╨/└┤╫╘╣1⁄2╓┌║┼í╛┬φ┐╦╩2╛▌═°í┐_2011.docx]

各位代表:
        现在，我代表市人民政府，向大会作工作报告，请予审议，并请市政协委员和其他列席人员提出意见。

**一、2010年工作回顾**

        过去的2010年，是“十一五”的最后一年，也是我们砥砺奋进、克难攻坚，保持经济平稳较快发展、实现“十一五”圆满收官的一年。一年来，在省委、省政府和市委的坚强领导下，全市上下坚持以邓小平理论和“三个代表”重要思想为指导，认真贯彻党的十七大和十七届三中、四中、五中全会精神，深入贯彻落实科学发展观，围绕加快建设现代国际旅游城市这一目标，紧扣加快转变经济发展方式这一主线，强力推进转型发展、开放发展、绿色发展、和谐发展，大力推进“十大工程”和“四区”建设，锐意进取，扎实工作，圆满完成了市五届人大五次会议确定的各项目标任务。
        **（一）经济保持较快增长**。初步核算，全市生产总值309.3亿元，同比增长13.1%，增速创1998年以来新高；财政收入44.3亿元，增长41.8%，增速居全省第一位；社会消费品零售总额126亿元，增长19%；固定资产投资454.3亿元，增长34.3%；完成“十大工程”项目投资312.8亿元，增长39.7%；完成省“861”计划项目投资242.8亿元，增长58.8%；争取国债项目378个，到位资金5.6亿元。过去的一年，我市重大项目和政策争取，实现了历史性突破，京福高铁黄山段已全面开工建设，皖赣铁路扩能改造芜湖至黄山北站段通过立项审批，黄杭快铁可行性研究报告通过专家评审，高铁歙县北站增设争取成功；国家服务业综合改革试点获得批准；黄山口岸扩大开放通过国家验收，实现对外国籍飞机开放；新安江流域生态补偿机制试点正式启动。这些重大项目和重大政策的实施，为“十二五”乃至今后一个时期的发展奠定了一个更为坚实的基础。
        **（二）结构调整步伐加快**。三次产业结构为12.7∶44.1∶43.2，其中二产同比上升3.5个百分点。农村经济平稳发展，全年实现农业增加值39.3亿元，增长4.7%；规模以上农产品加工企业181家，实现营销收入71.1亿元，增长40.6%，其中超亿元企业达10家；茶叶产量、产值继续保持全省第一，茶业综合产值达46亿元，增长29.6%，8家企业跻身全国茶业百强。工业经济全面提速，完成规上工业增加值101.6亿元，增长29.2%，增速居全省第三位，全年净增规上企业125家；完成技改投入63.5亿元，是“十五”总和的1.1倍；高新技术产业总产值突破90亿元，增长32.8%。全市园区完成固定资产投资86.4亿元、新增开发面积10.5平方公里、实现工业总产值231亿元，三项指标增速均超过50%；市经济开发区财政收入突破1亿元；歙县北岸经济开发区获省政府批准筹建。旅游经济快速发展，全年接待游客2544.7万人次，增长19.9%，其中入境游客突破百万大关，增长19.7%；旅游总收入202.1亿元，增长20.2%；旅游创汇3亿美元，增长23.4%。屯溪老街特色街打造、徽州府衙修复、西递•宏村旅游综合提升、中法乡村旅游合作等重点旅游项目快速推进，卧虎高尔夫、秀里影视村等建成运营。我市荣获中国最佳休闲城市、中国特色休闲城市、最中国旅游城市、十大中国最佳旅游目的地、中国休闲节会创新奖、中国休闲旅游营销创新奖等殊荣，并成为全国首个旅游服务标准化示范市。黄山风景区作为全国首家单位荣获世界旅游目的地管理奖，被评为全国首批数字化景区，荣获旅游景区创新奖，黄山风景区管委会作为亚洲唯一单位加入全球可持续旅游委员会，黄山旅游集团加入世界旅游业理事会并跻身全国旅游集团20强。
        **（三）改革开放成效显著**。创新举办第十四届中国黄山国际旅游节暨徽文化节，成功举办国际山地车节、国际登山大会、中国黄山（国际）风光摄影大展、中国黟县国际摄影节和第六届中国黄山茶叶暨名优农产品博览会等系列重大活动，在日本、韩国、法国和香港、深圳等地举办了系列旅游推介和招商等经贸活动，与法国尼斯市、南非开普敦市签订友好合作协议，成为安徽唯一的长三角世博体验之旅示范点城市。全年实现外贸进出口总额3.3亿美元，增长40.6%；引进内资300.8亿元，实际利用外资1.7亿美元，分别增长36.5%和20.7%；新签工业招商项目315个，协议投资额176.3亿元，分别增长16.2%和104.1%，其中亿元以上39个，是新签亿元以上工业项目最多的一年。各项改革纵深推进。筹措改制资金1.1亿元，全面完成市直41户国企改制任务。行政管理体制改革、农村综合改革稳步推进，“一事一议”财政奖补试点工作全面推开，农村土地整治整村推进工作取得初步成效，文化体制改革、基层医药卫生体制综合改革基本完成，市招标采购监督管理局、市徽文化产业投资公司和高铁新区开发投资公司组建成立。大力发展民营经济，分别有7家、16家和10家企业入选全省民营企业营销收入、纳税总额和出口创汇“百强”，我市被评为全省发展非公有制经济先进市。歙县、休宁县、祁门县被评为全省2009年度科学发展先进县。“黄山旅游”、“安徽恒远”被认定为“中国驰名商标”。持续超常规推进金融发展与服务，新成立农村合作银行和村镇银行4家、小额贷款公司4家，年末人民币贷款余额281亿元，比上年多增13.6亿元，增长33.1%，高出全省增幅10个百分点。
       **（四）城乡建设快速推进**。《黄山市城市总体规划（2008-2030）》修编获省政府批准实施。大力推进城市建设，中心城区完成投资87.6亿元，同比增长46%。新安江延伸段综合开发工程即将竣工，徽州文化艺术长廊和高铁新区正式动工，齐云大道、屯光大道拓宽改造工程全面完成，花山大桥和梅林大桥开工建设，全年新建、改建道路45条，新增道路总长32.2公里、道路面积42.7万平方米，新增供水管道40.3公里、供气管道21公里，新建绿化面积35万平方米，建成区绿化覆盖率达49.8%。加大拆迁安置区建设力度，全市完成投资11.3亿元，建成安置区12个、面积72.4万平方米。市城投公司6亿元债券成功发行，全年筹集资金25.2亿元，有力支持了城市建设与发展。大力推进区县城建设，全年完成投资79.8亿元，歙县城许大道、休宁县滨江路、黟县漳河流域综合整治、祁门县新城区、徽州区新城区、黄山区城市外环路等一批重点项目开工建设，城市框架进一步拉开、功能进一步完善、品位进一步提升，是历年来区县城建设发展最快、变化最大的一年。加大村镇建设力度，44个建制镇完成投资44.3亿元，增长26.2%。7个乡镇被评为国家级、省级环境优美乡镇，又有50个行政村达到新农村建设“十有”标准；新建乡镇综合文化站25个、农家书屋165个，591个自然村组实现广播电视村村通，新改建农村公路279.4公里，农村沼气覆盖率达22%，6.8万农村人口解决饮水安全问题。全市城镇化率达43.3%，提高1.6个百分点。
        **（五）民计民生持续改善**。城镇居民人均可支配收入15834元，超过全省平均水平，同比增长12.6%；农民人均纯收入6710元，高于全省1425元，增长17.7%。扎实开展“七保”工作，精心组织实施35项民生工程，共投入资金13.2亿元，惠及130万城乡居民，并在全省民生工程考核中名列前茅。城镇新增就业3.1万人、下岗失业人员再就业1.5万人，发放小额担保贷款5028万元，培训新型农民1.7万人，城镇登记失业率控制在3.9%以内。着力完善社会保障体系，制定出台城镇居民社会养老保险办法和工伤、失业保险市级统筹办法，养老保险实现省级统筹，城镇医疗保险实现全覆盖，全市五险参保人数达78.5万人；不断提高企业退休人员基本养老金、城乡低保标准和部分优抚对象以及五保对象待遇水平，城市低保生活补助水平和农村低保覆盖面继续位居全省前列，休宁县被列入国家新农保试点县；加大对低收入群体帮扶救助力度，城乡大病医疗救助达9.6万人次；采取有效措施缓解物价上涨对困难群众生活的影响，为城乡低保对象增发临时性生活补贴2649万元，城市低保物价补贴月人均超60元，是省定标准的3倍；加快保障性住房建设，发放廉租住房租金补贴9112户、1710.4万元，廉租房建设竣工10.4万平方米；全市“一卡通”发放涉农补贴4.6亿元，人均受益392元。高度重视深山区、库区和革命老区发展，扎实开展整村推进扶贫工程，投入扶贫资金5303万元，实施扶贫项目346个，7千人实现稳定脱贫。
        **（六）社会事业繁荣发展**。扎实开展国家技术创新工程试点工作，新增国家级高新技术企业5家、省级创新型（试点）企业8家、省级工程技术研究中心4家、国家和省重点新产品13个。免除义务教育阶段学生学杂费7108万元，发放中职学生资助款2741万元，完成中小学校舍安全工程加固改造和重建10.1万平方米，黄山职业技术学院正式设立并加紧建设，黄山民航职业技术学院获准筹建。加强文化遗产保护工作，“百村千幢”古民居保护利用工程完成投资10.3亿元，对537幢古民居实施了保护利用；百佳摄影点建设快速推进；新增中国历史文化名村2处，徽州传统木结构营造技艺入选联合国人类非物质文化遗产名录，“徽州文化生态保护实验区建设工程”项目入选文化部全国十大国家文化创新工程，“徽韵”入选国家旅游文化重点项目名录。《黄山市志》出版发行。完成77个村农民体育健身工程建设任务，广泛开展全民健身运动，在省十二运会、省五届残运会和省六届民运会上取得优异成绩。巩固完善城乡医疗卫生服务体系建设，投入3232万元完成472所乡镇卫生院、村卫生室和社区卫生服务机构建设；全面实行基层医疗机构药品价格零差率，药品价格平均降幅达40%以上；新型农村合作医疗参合率达95.3%。人口计生工作位居全省前列，低生育水平继续保持稳定，人口自然增长率控制在3.5‰以内。认真开展第六次全国人口普查。组织实施绿色质量提升行动，植树造林5.5万亩，新封山育林8万亩，绿色长廊线路绿化82.1公里，除险加固病险水库59座，综合治理水土流失面积90平方公里，省政府下达的“十一五”节能减排任务全面完成。加大耕地保护力度，新增耕地面积3359亩。制定出台加快推进城乡社区建设意见。加强食品药品安全监管，深入开展“安全生产年”活动，亿元生产总值安全生产事故死亡人数较上年下降13%。“五五”普法顺利完成。深入开展矛盾纠纷排查化解，加大信访积案化解力度，全市信访形势平稳、总量持续下降。加强社会治安综合治理，严厉打击各类违法犯罪活动，确保了全市社会稳定。国防动员、民兵预备役、人民防空和双拥优抚安置工作不断加强，被评为全国征兵工作先进市。妇女、未成年人、老年人、残疾人权益得到较好保障，新闻出版、文学艺术、哲学社会科学以及粮食、农机、物价、档案、气象、地质、地震和民族、宗教、侨务、对台等工作取得新的成绩。
        **（七）政府自身建设不断加强**。在加快发展的过程中，我们始终高度重视政府自身建设。扎实开展学沈浩创先争优活动，着力抓好“四大整治”，进一步提高科学发展的能力和水平。坚持依法行政，严格依照法定权限和程序履行职责，大力推进政务公开和政府信息公开，全面完成新一轮市县政府机构改革。加强行政服务中心建设，市政务大厅全年受理各类行政审批及服务事项10.98万件，提前办结率达99％。继续开展效能、政风、行风“三位一体”考核评议，办好“政风行风热线”和“市民连线”，受理群众和企业来信来电6826件。自觉接受市人大及其常委会的法律监督、工作监督和市政协的民主监督，进一步密切与工会、共青团、妇联等人民团体的联系，完善重大问题集体决策、专家咨询、社会公示和听证制度，认真听取各方面的意见和建议，推进决策科学化、民主化，市政府系统承办的461件人大代表建议和政协提案全部办结。严格落实党风廉政建设责任制和《廉政准则》各项规定，认真开展工程建设领域专项治理，切实解决损害群众利益的突出问题，加强审计监督和行政监察，严肃查处了一批违法违纪案件。各位代表！
        回首极不平凡的“十一五”，我们成功战胜了历史罕见的自然灾害，有效应对了金融危机的严重冲击，克服了资金、土地、环保等因素制约，破解了发展中的各种难题，胜利完成了“十一五”规划主要目标和任务，经济社会发展取得了令人鼓舞的巨大成就。
        **过去的五年，是我市综合实力提升最快的五年**。全市生产总值由158.6亿元增长到309.3亿元，年均增长11.8%，人均生产总值突破3000美元。财政收入由12.6亿元增长到44.3亿元，年均增长28.5% ，比“十五”增速快15.7个百分点，比全省平均水平高2.8个百分点。累计完成固定资产投资1435.5亿元，是“十五”的4.6倍，年均增长31.7%。全市三次产业结构由16.3∶35.6∶48.1，调整为12.7∶44.1∶43.2，第二产业上升了8.5个百分点，经济增长的稳定性、协调性和可持续性明显增强。
        **过去的五年，是我市改革开放成效最为显著的五年**。农业税全面取消，国企改革、集体林权制度主体改革顺利完成，行政管理体制、金融体制等改革不断深化，徽州文化生态保护实验区批准设立，皖南国际旅游文化示范区成为我省重要的区域发展战略。成功举办和承办纪念邓小平同志“黄山谈话”三十周年、第四届中国国际徽商大会等系列重大活动，与瑞士因特拉肯市结为友好城市，与韩国大邱市东区、瑞典瓦拉市交流合作持续发展。五年累计引进内资877.5亿元，实际利用外资6.2亿美元，分别是“十五”的7.4倍和4.3倍，其中引进工业项目1310个，到位资金115亿元，发展的活力和动力不断增强。
        **过去的五年，是我市城乡面貌变化最大的五年**。五年累计完成交通建设投入130.1亿元，是“十五”的1.7倍。合铜黄高速、黄塔（桃）高速建成通车，黄祁高速、黄绩高速开工建设，新改建农村公路4015公里，100%的乡镇和99.4%的行政村实现通沥青（水泥）路；黄山机场完成4D级改造，高铁建设拉开帷幕。五年累计完成城镇固定资产投资627.3亿元，全市城镇化率由35.1%提高到43.3%。中心城区和区县城建成区面积五年分别扩大了10平方公里和19平方公里，中国徽州文化博物馆、市游泳馆、全民健身馆、美术馆等一批公共设施建成使用。新农村建设累计投入资金近100亿元，165个村被列入省“千村百镇”示范村镇，202个行政村达到“十有”标准，城乡面貌焕然一新。
        **过去的五年，是我市人民群众所得实惠最多的五年**。五年累计投入民生工程资金31.8亿元，解决了一批事关群众切身利益的突出问题。社会保险五大险种全面实施，城乡免费义务教育全面实行。五年城镇新增就业7.8万人、下岗失业人员再就业5.3万人，连续5年提高企业退休人员养老金待遇。为1.2万户家庭解决了廉租住房或住房补贴。累计投入资金2亿元，完成了200处地质灾害危险点治理，2.4万人消除了地质灾害威胁。五年投入扶贫资金4.4亿元，贫困村农民人均纯收入由2005年的1693元，提高到2010年的5564元，34192人实现稳定脱贫。精神文明和民主法治建设不断加强，群众安全感连续五年位列全省前三名，跻身全国社会治安综合治理先进市行列。
        **过去的五年，是我市整体形象大幅提升的五年**。先后荣获了中国旅游竞争力百强城市、最佳国际休闲城市、世界特色魅力200强城市、中部最佳投资城市、中国外贸竞争力百强城市等殊荣，被评为国家园林城市、国家节水型城市、全国无障碍建设先进城市、全国双拥模范城和安徽人居环境奖城市、省首届文明城市，黄山的美誉度和影响力进一步扩大。
        各位代表！五年的励精图治，五年的艰苦创业，五年的开拓奋进，今日的黄山已经跨上了一个新的台阶，站在了一个新的起点，迈入了一个新的阶段。当前，全市上下建设现代国际旅游城市的思想更加统一、目标更加明确，加快“四区”建设、推进“十大工程”的支撑更加坚实、措施更加有力，人心思干、人心思上的精神状态更加饱满、工作干劲更加高涨，黄山必将创造新的辉煌，必将书写新的更加壮丽的篇章！
        取得的成绩令人振奋，积累的经验弥足珍贵。所有这一切，得益于省委、省政府和市委的正确领导，得益于市人大、市政协的监督支持，得益于全市人民的共同努力。在此，我代表市人民政府，向全市广大工人、农民、知识分子、干部和离退休老同志，向驻黄解放军指战员、武警官兵和政法干警，向各民主党派、各人民团体和各界人士，致以崇高的敬意！向关心、支持黄山现代化建设的中央、省驻黄单位和海内外友好人士，向在我市创业的投资者、建设者，表示衷心的感谢！
        在肯定成绩的同时，我们也清醒地看到，我市经济社会发展中还存在不少的困难和问题，主要是：经济总量小、实力弱，牵动性强的大项目、带动力强的大企业少，产业结构层次还较低；城乡发展还不平衡，中心城市带动力还不强，深山区、库区经济发展还相对滞后；居民收入和社会保障水平还不高，就业、教育、医疗等方面还存在一些薄弱环节，生态保护、防灾抗灾、安全生产和维护稳定的任务仍然十分繁重；政府少数部门服务意识不强、工作作风不实，机关工作效能和依法行政水平还需进一步提高。对此，我们将高度重视，在今后的工作中努力加以解决，不辜负全市人民的期望和重托。

**二、“十二五”发展的指导思想和奋斗目标**

        未来五年，是黄山不可多得的重要战略机遇期，也是黄山大有作为的黄金发展期。国内外产业转移深入推进，泛长三角区域合作不断深化，我市产业升级的条件更加有利；国家坚持扩大内需战略，实施区域发展总体战略，推动经济布局逐步从沿海向内陆延伸，我市得到的政策支持将更加有力；国家服务业综合改革试点在我市全面展开，皖南国际旅游文化示范区建设深入推进，徽州文化生态保护实验区加快建设，为我市加快发展提供了重要平台；工业化、城镇化步伐的加快，持续投入的累积效应不断释放，黄山经济发展的内生动力显著增强。我们必须把握一切有利条件，调动一切积极因素，努力保持经济社会又好又快发展势头，掀开黄山建设现代国际旅游城市的崭新画卷！
        根据《中共黄山市委关于制定国民经济和社会发展第十二个五年规划的建议》(以下简称《建议》)，我市“十二五”发展的指导思想是：以邓小平理论和“三个代表”重要思想为指导，深入贯彻落实科学发展观，以科学发展为主题，以全面转型、加速崛起、富民强市为主线，大力实施服务业提升战略、工业支撑战略、城乡统筹战略、开放创新战略、绿色发展战略和人才强市战略，持续推动转型发展、开放发展、绿色发展、和谐发展，强力推进“四区”建设，加快构建“四大体系”，为实现经济繁荣、人民富足、生态良好的发展目标，为全面建设小康社会，为早日建成现代国际旅游城市奠定坚实基础。
        主要目标是：努力实现“五个翻番”，就是到2015年地区生产总值、财政收入、累计固定资产投资、城镇居民人均可支配收入、农民人均纯收入比2010年翻一番以上；努力实现“五个突破”，就是旅游总收入突破500亿元、工业增加值突破300亿元、文化产业增加值突破100亿元、城镇化率突破50%、人均GDP突破6000美元。与此同时，文化建设、社会建设以及生态文明建设得到全面加强。
        在市委《建议》的指导下，市政府编制了《黄山市国民经济和社会发展第十二个五年规划纲要（草案）》，提请本次大会审议。
        我们相信：
        ——未来五年，伴随区域综合交通枢纽的建成，重大基础设施的完善，黄山连南接北、承东启西的区位优势，生态优越、资源卓越的后发优势日益凸显，将迈入厚积薄发、加速崛起的新阶段。
        ——未来五年，伴随现代服务业综合改革试点的深入推进，新型工业产业体系的加快构建，黄山的特色发展之路渐入佳境，综合经济实力显著增强，将呈现高增长与高质量并重、稳定性与成长性并行的发展态势。
        ——未来五年，伴随新一轮城市总体规划的加快实施，新农村建设的全面进步，黄山的国际化水平明显提升、地域化特征更加鲜明，徽风绵远的诗意田园与古韵幽香的时尚城市交相辉映，一座城乡互动、错位发展、独具魅力的现代国际旅游城市将闪亮登场。
        ——未来五年，伴随绿色质量提升行动的扎实推进，新安江流域生态保护体系的逐步完善，黄山的生态环境更为优越，绿色发展深入人心，我们将为拥有这方碧水蓝天、绿地青山而倍感欣慰和自豪。
        ——未来五年，伴随社会事业的繁荣发展，创业环境的不断优化，一切实干兴业、富民强市的活力将竞相迸发，一切锐意创新、勇于创优的源泉将充分涌流，勤劳智慧的黄山人民将迎来物质更丰裕、文化更丰富、社会更和谐的美好新生活！

**三、2011年主要工作**

        2011年是“十二五”开局之年，做好今年各项工作，意义重大。综合考虑，今年经济社会发展的主要预期目标是：地区生产总值增长12%；财政收入增长18%；全社会固定资产投资增长20%；外贸进出口总额增长20%；社会消费品零售总额增长20%，居民消费价格涨幅控制在4%左右；城镇居民人均可支配收入、农民人均纯收入均增长13%；城镇登记失业率控制在4%以内；人口自然增长率控制在4.5‰以内；万元生产总值能耗、主要污染物总量控制在省政府下达目标以内。上述目标是积极进取、自我加压的目标，同时也是通过努力、完全可以实现的目标。在实际执行中，我们将坚持能快则快、好中求快，努力实现又好又快发展。
       重点抓好以下九个方面的工作：
       **（一）努力扩大有效投入**
        继续坚持把扩大有效投入作为加快黄山发展的主动力来抓，加大项目建设力度，提高建设资金保障水平。
        **强力推进重点项目建设**。突出抓好595个“十大工程”项目和154个(第一批)省“861”项目，分别完成投资373亿元和84.4亿元，确保152个项目建成运营。全力推进京福高铁工程建设，黄绩高速竣工通车，黄祁高速完成路基主体工程并铺筑路面，尽早开工黄杭快铁、皖赣铁路扩能改造、黄山北站和歙县北站站场建设，积极推进歙县界牌岭至黟县渔亭一级公路改建项目。建成徽州500千伏、芳村220千伏和忠塘110千伏输变电工程。加大黄千高速、黄池城际铁路和月潭水利枢纽等重大项目推进力度，力争一批重大项目列入国家和省里计划。精心谋划和编制一批基础设施、优势产业和战略性新兴产业项目，力争储备重点项目超400个、总投资超4000亿元。全面落实项目推进工作机制，优化项目建设环境，加强项目调度督查，确保重点项目早开工、快建设、多竣工。
        **切实增强融资保障能力**。以推进项目建设为重点，持续超常规推进金融发展与服务，力争全市新增贷款100亿元（全口径）。确保新农村建设(百村)示范项目8000万美元世行贷款列入国家计划。抓住国家加大保障性住房、水利设施、农业基础设施、教育卫生基础设施、节能减排和生态环境等方面投入的机遇，力争国债资金突破5亿元。抓好政府融资平台建设，力争全市直接融资达到100亿元，中心城区建设融资超过30亿元。深化与国开行、农发行及其他商业银行的新一轮合作，确保市城投公司间接融资超过18亿元，并力争发行二期企业债券。加强企业债券项目建设的资金管理，放大项目资金使用效益。支持和引导社会资本加大对项目建设投入。
        **（二）着力提升服务业发展水平**
        全面启动服务业综合改革试点工作，加快打造皖南国际旅游文化示范区核心区，加快推进徽州文化生态保护实验区建设。
        **强势扩张旅游经济**。继续围绕“六个转变”，切实抓好81个旅游综合开发提升工程项目建设，确保完成投资74.9亿元。加快黄山风景区打造国际精品旅游景区进程，全力推进南大门整治改造和北大门现代旅游服务业集聚区建设，全面完成西海饭店改造、南大门立体停车场二期、西海大峡谷地轨缆车等项目建设。加快推进屯溪老街、东黄山、呈坎、潜口、唐模、棠樾、雄村和牯牛降等重要节点建设，力争西递、宏村进入5A景区行列，新增4A景区3家，全市3A以上景区总数达到44家。加快完善旅游基础设施和接待设施，开工建设11家高星级酒店、5个自驾车营地，建成旅游信息咨询点20个，兴建大型旅游超市。大力发展新型旅游业态，打造雨润、太平湖、奇墅湖、新徽天地、纳尼亚小镇等高端休闲度假基地，加快新安江延伸段水上旅游、齐云山养生旅游和牯牛降生态旅游开发。持续推进乡村旅游发展，新增农家乐接待户300户，继续抓好中法乡村旅游合作示范项目。编制户外旅游发展规划，建立健全户外旅游的安全保障体系，推出5条以上精品户外旅游线路，规范、促进户外旅游加快发展。强化旅游整体对外营销，持续推进旅游“三进”，全面落实境外旅游合作协议。着力拓展航空市场，增开黄山至首尔定期航线，恢复和新开黄山至丽江、厦门、西安、昆明和黄山至香港、日本、新加坡等境内外航线和包机，加密现有航线航班，积极争取与台湾直航，力争黄山机场年旅客吞吐量突破50万人次。强力推进“智慧黄山”精品旅游信息化工程，鼓励支持乡村旅游网站建设。加大旅游联合执法力度，推进旅游诚信体系建设。全年接待游客2950万人次，增长16%，旅游总收入245亿元，增长21%；其中，接待入境游客131万人次，增长25%，旅游创汇3.8亿美元，增长25%。
        **大力发展文化产业**。继续实施文化产业精品打造工程，确保24个重点项目完成投资31.7亿元。加大文物和非物质文化遗产保护力度，完成“百村千幢”古民居保护利用二期工程建设任务，扎实推进徽州文化艺术长廊、屯溪老街、徽州古城、黄山国际健康产业园和齐云山生态文化旅游区等园区和基地建设。大力发展文博文化、摄影书画、演艺影视、红色文化、海派文化、宗教文化、民俗文化等新型文化业态，提升“百村千幢”古民居、百佳摄影点、博物馆集群、东黄山知青部落、新四军军部旧址、黟县梓路寺、秀里影视村等项目综合效益。进一步打响“徽韵”品牌，打造徽州府衙、元一全景剧院、花山谜窟激光秀、“宏村阿菊”等一批高端文化设施和演艺产品。进一步扩大黄山国际旅游节、山地车节、黄山温泉文化旅游节、登山大会、摄影节等节庆赛事活动对外影响力，努力打造独具特色的节庆文化产业和体育赛事产业。扶持引导旅游文化商品研发和民间工艺传承，打造徽州四雕、文房四宝、徽派盆景等一批特色旅游文化商品生产基地，力争年销售额突破10亿元。支持文化龙头企业做大做强，培育创意、传媒、动漫、信息服务等新兴文化企业，实现文化产业增加值增长30%以上。
        **丰富发展其他服务业**。改造提升商贸流通等传统服务业，积极发展连锁经营等大型商业业态，力争世界500强零售集团入驻黄山，世界知名零售集团大润发黄山店建成开业。支持市内品牌商贸企业走出去开拓市场。大力发展专业市场和区域性市场，新建1个交易额超亿元的专业批发交易市场。创新发展徽菜产业。扎实推进“万村千乡市场工程”和社区“双进工程”，继续开展家电下乡、“以旧换新”工作，不断繁荣城乡消费市场，全年实现社会消费品零售总额151亿元。结合第二居住地建设，发展多种形式的景观房产、度假社区，引导合理的房地产消费。加快雨润国际会展中心等会展设施建设，培育繁荣商务会展产品，积极举办承办具有国内外影响力的会议、展览。大力发展金融、保险、证券、信托业，着力发展会计、审计、评估、法律服务等中介服务业，积极发展第三方物流服务机构。推进家政服务业发展，初步构建覆盖中心城区和区县的家政服务网络。
        **（三）做大做强工业经济**
        深入推进园区提速增效工程，不断扩张工业规模，强化科技支撑，确保规模以上工业增加值增长30%。
        **大力扩张优势产业**。制定实施优势产业培育行动计划、大企业大集团培育行动计划，进一步加大机械电子等七大优势产业发展力度，积极发展电子信息等六大新兴产业。支持永佳集团、兴乐铜业、金马股份、皖南机床、黄汽电公司、嵊峰袜业、黄山工业泵、黄山电器等重点骨干企业规模扩张、加快发展，支持日普电动车、中显微电子等重点工业项目尽快竣工投产，支持一批中小企业迅速做大做强，确保新增规上企业60户(新口径)。加强对重点行业、骨干企业的运行监测和协调服务，帮助企业提高效益和质量。继续安排市优势产业发展专项资金5000万元，筹集2亿元设立战略性新兴产业发展引导资金和风险投资引导基金，积极发展多种所有制形式的融资性担保机构，力争担保金额超过22亿元。支持上市公司再融资，加快拟上市企业的储备、改制和上市辅导工作，进一步拓宽企业直接融资渠道，确保5户企业完成股改、2 户企业进入辅导期、1户企业实现上市申报。
        **大力发展园区经济**。按照“三个围绕、一个不上”和“三个集中”的要求，制定实施百亿园区培育行动计划，全力推进园区提速增效工程194个重点项目建设，力争全市园区新增开发面积8平方公里，竣工项目170个，完成投资105亿元，实现工业总产值290亿元，税收收入10亿元。加大园区工业招商力度，力争引进一批节约集约用地的大项目、好项目。支持市经济开发区实施“411翻番”发展战略，加大光伏、汽车配件、LED等新兴产业项目招商力度，确保新签项目40个，开工和投产项目各30个，固定资产投资、工业总产值分别达到30亿元和25亿元。加快市循环经济园和北岸经济开发区建设力度，支持八个产业集群专业镇发展特色产业园，力争屯溪九龙低碳经济园区列入省级开发区。
        **大力推进自主创新**。抓住我省开展国家技术创新工程试点的机遇，改造提升传统制造业，积极发展新兴产业。着力抓好120个投资超千万元重点技改项目建设，力争全市完成工业技改投资80亿元以上，高新技术产业总产值突破100亿元。加快车用仪表及电器、绿色包装材料两个省级高新技术产业基地建设，力争永新股份进入国家级高新技术特色产业基地行列。建立健全推进企业技术创新的配套政策和措施，支持企业实施重点科技项目、建设研发中心和专业技术服务平台，力争新增国家高新技术企业3家，新增省级创新型企业2家。以新技术研究、应用和推广为重点，全面推进与高校院所产学研合作，加快市科技孵化器建设，力争入孵企业达45户。大力实施商标战略和知识产权战略，鼓励技术创新和发明创造，切实提高专利的产品转化率。
        **（四）扎实做好“三农”工作**
        认真落实“八个一批”的要求，深入推进新农村建设巩固拓展工程，着力促进农业增效、农民增收，力求新农村建设取得新进展。
        **大力发展现代农业**。坚持在工业化、城镇化深入发展中同步推进农业现代化。加大产业基地、龙头企业、知名品牌和重大项目建设力度，进一步做大做强主导产业，产值超亿元龙头企业达到12家以上。继续推进生态茶园“双十”工程，建成高效生态茶园5万亩，办好黄山茶叶暨名优农产品交易会系列推介活动，力争茶业综合产值达到52亿元。进一步发展蚕桑、贡菊、花卉苗木和养殖业，新建蔬菜基地8000亩。抓好黄山区现代农业综合开发示范区建设。加快实施林业产业化“4646”工程，调整林种树种结构，大力发展“一竹四果”产业，新建油茶基地2.9万亩，着力培育一批林业名牌产品和龙头企业，力争全市林业总产值突破80亿元。做好绿色、有机、无公害食品认证和地理标志产品保护工作。推进省级第七批农业标准化示范区项目建设，加强重大植物病虫害统防统治和动物疫病防控，加强农产品质量安全监测体系建设，确保我市农产品质量安全。
        **深入推进新农村建设**。坚持把加强农村基础设施建设、改善农村公共服务作为新农村建设的重要内容。加强农村公路建设，完成农村危桥改造20座，继续实施农村清洁工程，深入开展村庄整治，改善农村生产生活条件。完善农业科技服务体系，加大农业适用技术应用推广力度，新增新农村建设科技示范乡镇10个。抓好农村薄弱学校建设。加强农村公共文化设施和信息化建设，完成35个乡镇综合文化站、337个农家书屋、30个村级农村综合信息服务站和广播电视村村通建设任务。加强农村公共卫生服务和基本医疗体系建设，新型农村合作医疗参合率保持在95%以上。加强农民技能培训，积极引导农民工返乡创业，拓宽农民增收渠道。加快新农村“十有”标准示范村建设，新增达标村和基本达标村各50个。
        **不断深化农村各项改革**。认真贯彻中央一号文件精神，加快水利改革发展，加大中小河流治理力度，加固除险小型病险水库29座，新建抗旱机井1000口，抓好新一轮农村饮水工程建设。全面开展第一次全国水利普查工作。大力发展县域经济，切实抓好省扩权强镇试点镇建设。深入推进农村综合改革，进一步完善为民服务全程代理制。做好农村义务教育债务清理化解后续工作，防止产生新的债务。把维护农民合法权益放在首位，充分尊重农民意愿，规范开展农村土地整治整村推进试点，稳步推动移民下山出库和异地搬迁安置。深化农村金融改革，组建农村商业银行1家、村镇银行2家，实现区县小额贷款公司全覆盖。做好政策性农业保险试点工作。全面深化、整体推进集体林权制度改革，推进国有林场改革，加强林业要素市场建设，支持开展林权抵押贷款担保业务，争取开展国家和省级政策性森林保险试点，力争新建林业专业合作社20个以上。
        **（五）强力推进城镇建设**
        以新一轮城市总体规划为引领，按照“惠及百姓、提升形象、完善功能、促进发展”的要求，扎实推进城乡建设管理提升工程，力争城镇固定资产投资突破260亿元，全市城镇化率提高1.8个百分点。
        **继续抓好中心城区建设**。计划安排政府投资项目40个、社会投资项目60个，完成固定资产投资106亿元以上，增长32.5%。加快新安江延伸段综合开发进程，续建完成新安江湖边至花山段6.3公里两岸道路、景观绿化、亮化等工程，启动两岸10平方公里区域、4500亩可开发建设用地规划建设。加快实施以拓宽火车站至横江桥过境段、西海路为重点的市政道路改造升级工程，实施草市至北海路、横江桥至梅林大道段的景观、亮化、供排水、供气改造及沿线风貌整治，形成篁墩至屯黄高速1号口长达18公里、横贯东西、顺畅新老城区的城市快速过境景观大道。开工建设文峰桥，续建花山大桥、梅林大桥，改造横江桥，新建上草市路、桑园路、汉沙路、码头路、梅林南路、西区二号路、高职南侧区间路，贯通新园西路，启动建设仙人洞南路。安排投资500万元，对5条小街、小巷和5个小区进行改造治理。继续完善给排水、消防、电力、电讯、无障碍等市政公用设施。新增和延伸5条公交线路，更新公交车辆40辆以上，进一步提升公交服务和管理水平。完成投资20亿元，全力推进高铁新区“一河三桥七路”改造与建设，形成两横两纵主干道路网，拉开高铁新区总体框架；启动场站和站前广场、公交站、汽车站、停车场、配套管网等基础设施建设。全力推进徽州文化艺术长廊建设，新建百师宫、假日客栈项目，全面完成入口形象区、凤霞安置区、项目区主干道及其景观建设，加大招商开发力度，确保年内见成效、出形象。抓好总建筑面积278.7万平方米、总套数18129套的25个安置区建设，完成重点项目征迁80万平方米。
        **加快区县城和小城镇建设步伐**。坚持以规划为龙头，加紧完善重点区域控制性详规和各类专项规划，并确保规划严格执行。加快区县城综合改造建设，突出抓好屯溪区江南新城、黄山区西海路、徽州区新四军军部旧址及周边环境整治、歙县交通旅游集散中心、休宁县滨江新区、黟县漳河滨水景观带、祁门县新城区等项目，推进区县城基础设施配套升级。强化引导扶持，重点抓好20个基础好、潜力大的特色城镇建设和发展，力争全市区县城、建制镇分别完成投资100亿元和56.4亿元。
        **加大城市管理和创建力度**。坚持专项整治与长效管理并举，针对薄弱时段、薄弱地段和薄弱环节，突出抓好市容环境综合整治，大力推行市容精细化管理，重点实施环卫基础设施配套、旅游公厕改造升级、户外广告规范提档、渣土车辆规范管理、数字化城管信息系统5大项目工程，大力推进依法拆违控违工作，强化小区物业管理和示范小区建设，切实解决市民和游客反映强烈的突出问题。深入实施城市文明提升工程，广泛开展多种形式的文明创建活动，不断提高市民综合素质，努力争创全国创建文明城市工作先进城市。
        **（六）切实加强生态建设**
        牢固树立绿色、低碳发展理念，大力推进生态环境保护建设工程，建立完善可持续发展的生态经济体系和消费模式，争创“中国人居环境奖城市”。
        **推进节能减排和资源节约利用**。严格执行节能减排工作责任制。强化工业企业环境监管，继续实施强制性清洁生产审核和水污染物排放许可制度，严格整治涉铅企业，加强工业大气污染源防治。加快城镇污水处理厂及配套管网建设，36个镇、375个村分别建成垃圾集中处置和收集转运系统。加强固体废物和辐射管理，综合整治城市油烟、噪声扰民问题。强化21户年耗能3000吨以上标煤企业的监管，落实各项综合节能措施。完善建筑节能闭合管理体系，推广节能保温材料和利用太阳能。抓好交通运输节能，全面推动公共机构、宾旅馆等领域节能，强化亮化设施管理工作。加快天然气利用工程建设，新建农村户用沼气1000口。推进循环经济园区建设，做好资源综合利用认定企业申报工作，再争创1～2家省级循环经济示范单位。严格执行政策，坚决纠正土地违法行为。加快闲置土地清理，落实建设密度、投资强度“双控”标准，推进土地节约集约利用。
        **加大生态环境保护和建设力度**。投资3.6亿元，实施437个、4.6万亩的绿色质量提升点建设。继续抓好巩固退耕还林、林业综合开发、松材线虫病三道防线、森林重点火险区治理等林业生态工程项目，完成人工造林6万亩。综合治理水土流失90平方公里，力争所有区县达到地质灾害群测群防“十有县”目标。强化河道管理，有效保护饮用水源地，新增城镇年供水能力2亿立方米，再解决6.8万农村人口饮水安全问题。全面启动新安江流域生态补偿机制试点。加快歙县省级可持续发展实验区建设。创建1个省级生态县、5个和10个省级以上生态乡镇、生态村。全市地表水水质达标率、空气质量优良率继续保持100%。 
        **(七)继续推进改革开放**
        以改革促创新，以开放促发展，进一步激活内力、集聚外力，力争全年实际利用外资增长10%、引进内资增长25%，民营经济增加值增幅有新的提高。
        **扎实推进重点领域和关键环节改革**。深化行政管理体制改革，完善招标采购监管体制，扩大统一招标采购范围，建立市级和区县公共资源要素交易市场。深入推进事业单位人事制度改革，完善事业单位岗位设置管理新机制。深化收入分配制度改革，扎实推进事业单位绩效工资实施工作。深入推进经营性事业单位转企改制。强化税收征管改革，建立健全科学的地方税收管理体系，加大税收征管和稽查力度，确保财政收入持续稳定增长。完善预算编制和执行管理制度，优化财政支出结构，重点加大对公益设施、民生保障、农村发展、社会建设、涵养税源等方面的投入。扎实开展中小企业信用体系试验区建设，创新信贷融资产品，积极探索土地承包权、集体建设用地使用权、知识产权、应收账款、股权、商标权等抵押或质押贷款。进一步放宽民间资本进入的行业和领域，加快产权多元化改造步伐，大力发展非公经济。
        **大力开展招商引资**。主动融入长三角，积极对接珠三角、闽三角，进一步完善招商引资工作机制，加大招商引资力度，力争全年引进项目500个以上，引进内资375亿元、外资1.9亿美元。坚持实行招商引资一把手负责制，完善考核奖励办法，调动各级各部门招商引资积极性，形成强大工作合力。按照转型发展要求，根据产业转移规律，修订完善招商引资政策，提升招商引资的质量和水平，新签工业、旅游、商贸项目比重提高到80%以上。加强招商人才队伍建设，科学编排重大招商引资项目库，深化与央企、省企和大型民企的战略合作，力争在徽商大会、中博会、厦洽会、高交会、皖台经贸活动等大型招商活动中取得更大成效，努力在引进大企业、大项目上实现新的突破。推行重点招商项目“四个一”工作制度、联席会议制度、督查督办制度，搭建招商引资高效服务平台，确保招商项目顺利落地。努力扩大对外贸易。认真落实外贸进出口政策，培育进出口大户，扩大进出口规模，力争全年实现外贸进出口总额3.9亿美元。调整优化出口产品结构，扩大农产品、纺织服装、精细化工、机械电子等优势产品和高新技术产品出口份额，抓好先进技术、关键设备和资源性原材料进口。引导企业实施品牌战略，加快质量管理体系认证，增强国际市场竞争能力。
        **（八）着力改善民计民生**
        始终坚持以人为本，切实保障和改善民生，让人民群众得到更多的实惠，保持社会和谐稳定。
        **巩固提升民生工程**。认真组织实施36项民生工程，投入总规模增加到16亿元。坚持群众满意标准，全面落实配套资金，强化工程建设质量，严格项目资金监管，夯实基层基础工作，不断完善民生工程长效化管理机制，推进民生工程与社会事业、社保体系建设的衔接融合，力争全市民生工程工作继续走在全省前列。
    **完善就业和保障体系**。实施更加积极的就业政策，统筹做好高校毕业生、农村转移劳动力、城镇就业困难人员的就业工作，新增城镇就业1.7万人。积极引导农民工就地就近就业。加快建立覆盖城乡居民的社会保障体系，加大社会保险征缴扩面力度，城镇五险参保人数达到79万人。加快城镇职工医疗保险市级统筹，抓好休宁县国家级新型农村社会养老保险试点工作。提高城乡低保、农村五保供养标准和城乡大病医疗救助水平。加强残疾人服务和保障体系建设，积极做好残疾人康复、就业和扶贫工作。加大保障性住房建设力度，新建廉租住房2700套、经济适用房1000套、公共租赁住房2200套，完成棚户区改造5000户。继续加大“菜篮子”工程建设力度，稳定和提高蔬菜自给能力。深入实施扶贫开发整村推进工程和产业扶贫，提升贫困村和贫困家庭自我发展能力。
        **加快发展社会事业**。推进义务教育均衡发展，优化教育资源布局，加快义务教育阶段标准化学校建设，完成中小学校舍安全工程建设任务。积极发展学前教育，切实缓解入园难问题。推进黄山职业技术学院和黄山民航职业技术学院建设，扩大电大办学规模，支持黄山学院加快创办综合性大学。开展全国科普示范县创建活动，实施科普惠农兴村计划。深入实施人才强市战略推进工程，大力培育黄山特色人才队伍。深化医药卫生体制改革。完成市突发公共卫生事件应急指挥系统建设，建成全市公共卫生应急物资储备库。积极传承新安医学，实施中医药“三名”、“三进”工程。加强食品安全监管，加快农村药品供应网和监督网建设。深入开展爱国卫生运动。进一步加强人口和计划生育基层基础工作，抓好流动人口服务管理，继续稳定低生育水平。大力开展全民健身运动。开工建设市档案馆，加快筹建市图书馆。统筹建设老年人和青少年活动设施。支持工会、共青团、妇联等群众团体依照法律和各自章程开展工作。切实维护妇女、儿童和老年人合法权益。
        **切实维护社会稳定**。全面落实安全生产责任制和责任追究制，着力构建“8+1”安全生产长效机制，推进“6100”行动计划，加大重点行业和领域安全生产隐患排查治理力度，严防重特大安全生产责任事故发生。扎实做好地质灾害防治和气象、地震等工作。启动“六五”普法，继续开展“江淮普法行”活动。坚持以群众工作理念统揽信访工作，强化信访工作责任制，加大矛盾纠纷排查化解力度，加强突发事件预警预防和应急处置体系建设，积极防范群体性事件。完善社会治安防控体系，深入开展打黑除恶专项斗争，严密防范和打击违法犯罪活动，确保人民生命财产安全，积极争创“长安杯”。推进和谐社区建设，加快社区管理体制改革，提升村务公开民主管理规范化建设水平。做好民族宗教、外事侨务和对台工作。深入开展双拥模范城（县）创建活动，抓好优抚安置、民兵预备役和人民防空工作，落实优抚安置政策，力争实现“全国双拥模范城”三连冠。
        **（九）不断加强政府自身建设**
        做好今年各项工作，开创“十二五”发展崭新局面，需要政府勇担使命、积极作为，不断加强自身建设，努力建设人民满意的服务型政府。
        **振奋精神，打造责任政府**。深入开展向杨业功、沈浩、张宁海学习活动，扎实推进创先争优，始终保持昂扬向上的精神状态，始终保持奋发有为的工作激情，集中精力抓发展，干事创业争一流。深入开展机关效能和作风建设，狠抓工作落实，切实解决一些制约发展的历史遗留问题，树立政府勤政务实的良好形象。落实行政首长负责制、工作责任制和责任追究制，确保令行禁止、政令畅通，切实提高政府执行力。继续办好“政风行风热线”和“市民连线”，深入开展“四大整治”和效能、政风、行风“三位一体”评议以及“百家企业评机关”活动，综合运用目标管理、考核督查、明查暗访、行政问责等手段，着力解决慢作为、不作为、乱作为等问题。
        **执政为民，打造亲民政府**。牢固树立群众观点，倍加珍惜人民赋予的权力，千方百计排民忧、解民难，让群众更多更好地共享改革发展成果。更加重视社会管理和公共服务，着力保障和改善民生，切实解决就业、教育、医疗、市场物价、社会保障、征地拆迁等方面人民群众最关心的问题。加大行政执法力度，严厉查处危害安全生产、食品药品安全、自然资源和环境保护、社会治安等方面的违法案件，维护公共利益和经济社会秩序。认真贯彻实施政府信息公开条例，全面推进政务公开和办事公开工作，提高政府的公信力。进一步加强行政服务中心建设，强力推进“应进俱进”、“两集中两到位”和基建项目“一表清”、工商注册登记“一表制”，为企业和群众生产生活提供优质、高效、便利的服务。
        **依法行政，打造法治政府**。牢固树立社会主义法治理念，切实提高运用法治思维和法律手段解决经济社会发展中突出矛盾和问题的能力。加强行政决策程序建设，把公众参与、专家论证、风险评估、合法性审查和集体讨论决定作为重大决策的必经程序，推进行政决策的科学化、民主化、法治化。完善行政决策风险评估机制，凡是有关经济社会发展和人民群众切身利益的重大政策、重大项目等决策事项，都要进行合法性、合理性、可行性和可控性评估。自觉接受人大及其常委会的法律监督、工作监督和政协的民主监督，办好人大代表议案、建议和政协提案，经常向人大报告、向政协通报重大事项，认真落实人大的决议决定和审议意见，注重采纳政协的调研成果和意见建议。广泛听取民主党派、工商联、无党派人士和各人民团体的意见，主动接受社会公众和新闻舆论监督。
        **廉洁自律，打造清廉政府**。认真落实《廉政准则》，坚持用制度管权、管事、管人。建立健全教育、制度、监督并重的惩治和预防腐败体系，认真执行反腐倡廉各项制度，切实加强廉政建设，深入开展反腐败斗争。加强对项目招投标、国有土地出让、产权交易、政府采购、政府投资、公务消费等监督，加强对扶贫救济、社保基金等社会公共性资金规范管理。以构建权力阳光运行机制为重点，加快推进电子政务和“数字监察”系统建设，充分发挥监察、审计部门的监督作用，加强对行政权力运行的监督和制约。

        各位代表！黄山美好的前景催人奋进，科学发展的征程任重道远！让我们在省委、省政府和市委的坚强领导下，高举中国特色社会主义伟大旗帜，深入贯彻落实科学发展观，团结和带领全市人民，开拓进取，扎实工作，全面完成今年的各项目标任务，加快建设现代国际旅游城市，努力开创黄山转型发展、开放发展、绿色发展、和谐发展的新局面，以优异成绩向中国共产党成立90周年献礼！
